# Full Tetragonal Phase Stabilization in ZrO_2_ Nanoparticles Using Wet Impregnation: Interplay of Host Structure, Dopant Concentration and Sensitivity of Characterization Technique

**DOI:** 10.3390/nano8120988

**Published:** 2018-11-28

**Authors:** Claudiu Colbea, Daniel Avram, Bogdan Cojocaru, Raluca Negrea, Corneliu Ghica, Vadim G. Kessler, Gulaim A. Seisenbaeva, Vasile Parvulescu, Carmen Tiseanu

**Affiliations:** 1 National Institute for Laser, Plasma and Radiation Physics, RO 76900 Bucharest-Magurele, Romania; claudiu.colbea@inflpr.ro (C.C.); radu.avram@inflpr.ro (D.A.); 2 Department of Chemistry, University of Bucharest, B-dul Regina Elisabeta, nr. 4-12, 030018 Bucharest, Romania; bogdan.cojocaru@chimie.unibuc.ro (B.C.); vasile.parvulescu@g.unibuc.ro (V.P.); 3 National Institute of Materials Physics, 405A Atomistilor Street, 077125 Magurele-Ilfov, Romania; raluca.damian@infim.ro (R.N.); cghica@infim.ro (C.G.); 4 Department of Molecular Sciences, Biocenter, SLU, Box 7015, SE-75007 Uppsala, Sweden; vadim.kessler@kemi.slu.se (V.G.K.); gulaim.seisenbaeva@slu.se (G.A.S.)

**Keywords:** ZrO_2_, tetragonal phase stabilization, average structure, local symmetry, wet impregnation, luminescence

## Abstract

Here, we show that wet impregnation of ZrO_2_ nanoparticles with 10% and 20% Eu oxide followed by thermal anneal in air above 500 °C produces full stabilization of the tetragonal phase of ZrO_2_ without evidencing any phase separation. The bare ZrO_2_ nanoparticles were obtained using three synthetic methods: oil in water microemulsion, rapid hydrothermal, and citrate complexation methods. The homogeneity of the solid solutions was assessed using X-ray diffraction, Raman spectroscopy, high resolution transmission electron microscopy, and advanced luminescence spectroscopy. Our findings show that wet impregnation, which is a recognized method for obtaining surface doped oxides, can be successfully used for obtaining doped oxides in the bulk with good homogeneity at the atomic scale. The limits of characterization technique in detecting minor phases and the roles of dopant concentration and host structure in formation of phase stabilized solid solutions are also analyzed and discussed.

## 1. Introduction

Zirconium oxide (ZrO_2_) is a well-established ceramic material where the physical and chemical properties depend strongly on the structural phase leading to a variety of applications [1,2]. Both tetragonal and cubic phases can be stabilized at ambient temperatures upon doping with trivalent ions such as Y^3+^ or lanthanides (Ln) [3,4]. Due to the facile doping of the Ln metals in ZrO_2_ lattice, there are many reports that describe the potential applications of Ln doped ZrO_2_ as dielectric film transistor [5], white light emitting diodes [6], catalysis [7,8], fuel cells [9], temperature sensor [10], oxygen sensor [11], dosimetry [12], photocatalyst [13], and bioimaging [14]. Among the Ln series, Eu is considered as an ideal dopant/stabilizer as the average structural properties of ZrO_2_ can be correlated with the local scale properties around Eu. As such, Eu shows distinct changes in the emission/excitation spectra and excited state dynamics with changes in the local symmetry with fingerprint emissions characteristic to monoclinic and tetragonal phases identified and extensively described [15,16,17,18,19,20]. The mechanism of tetragonal and cubic phase stabilization using lanthanide doping is well-established. According to Li et al. [4], the oversized aliovalent lanthanide metals are effective for the stabilization of tetragonal and cubic phases at room temperature via generation of the oxygen ion vacancies. To maintain its effective coordination number close to 7, as required by the (partial) covalent nature of the Zr–O bond, the ZrO_2_ lattice assumes a crystal structure, which offers an 8- coordination number (typically tetragonal or cubic structures) and simultaneously incorporates the generated oxygen vacancies into the lattice as the nearest neighbors to Zr^4+^ cations, and thus, next- nearest neighbors to the trivalent Ln.

So far, ZrO_2_ nanoparticles stabilized in the tetragonal/cubic phase by lanthanide doping are obtained using a multitude of synthetic approaches that include: microemulsion oil in water [18,19,21], hydrothermal/rapid hydrothermal [22,23,24,25], coprecipitation [26,27], sol-gel [28,29], Pechini process [30], sol-emulsion-gel [11,31,32], combustion synthesis [13,33,34,35,36], solar physical vapor deposition [37], and complex polymerization method [38]. To the best of authors’ knowledge, there is no study that investigates the effect of wet impregnation with a lanthanide oxide on the tetragonal/cubic phase stabilization of ZrO_2_ nanoparticles. Wet impregnation, otherwise a well-known method used for synthesis of heterogeneous catalysts, exposes the host oxide to a liquid containing the precursor of the dopant, which is then dried and heated in air. In the best scenario, the final result is a surface-doped oxide without formation of a separate phase of dopant oxide or a submonolayer of the dopant oxide on the host oxide [39]. In the case of ZrO_2_, homogenous doping in the bulk by wet impregnation with a high amount of lanthanide oxide typically needed for phase stabilization therefore seems highly improbable. Here, we investigate the effects of wet impregnation on the structural phase of ZrO_2_ nanoparticles by use of X-ray diffraction (XRD), Raman spectroscopy, transmission electron microscopy (HRTEM), and advanced luminescence spectroscopy. We show that wet impregnation of ZrO_2_ with 10% and 20% Eu oxide followed by thermal anneal in air above 500 °C leads to solid solutions of tetragonal phase that are homogenous at the atomic scale. The doping efficiency does not depend on the synthetic route used in synthesis of bare ZrO_2_ (rapid hydrothermal, oil in water microemulsion, or citrate complexation method), or surface area (that vary from 250–300 m^2^/g to few m^2^/g). The complementarity between the characterization techniques highlights the ability of luminescence to detect minor monoclinic phase disregarded by both X-ray diffraction and Raman spectroscopy. A comparison with another significant fluorite oxide, CeO_2_, highlights the key role played by the fluorite structure in the efficiency of “dissolving” the lanthanide oxides.

## 2. Materials and Methods

### 2.1. Nanoparticles Synthesis

Bare ZrO_2_ nanoparticles were prepared using three distinct synthetic routes: oil in water microemulsion (OW), rapid hydrothermal (RH), and citrate complexation (CIT) methods. Details on each of the method are published elsewhere [18,28,40]. These supports were impregnated with 10% Eu (in the case of OW and RH) and 20% Eu (in the case of CIT). A brief description of the synthesis procedures is however presented. For the microemulsion synthesis, the following precursors were chosen: Synperonic® 10/6, Zirconium (IV) ethylhexanoate (Alfa Aesar, Ward Hill, MA, USA), and Hexane (Merck, Whitehouse Station, NJ, USA). The employed microemulsion system was: water/Synperonic®10/6/hexane. An isotropic solution at 35 °C was obtained by mixing all the above-mentioned components. The maturation time was 48 h after the pH was adjusted to 11. The obtained precipitate was washed using a mixture of ethanol and chloroform (1:1). Zirconium nitrate (Alfa Aesar, Ward Hill, MA, USA ) was chosen as a precursor alongside citric acid (Alfa Aesar, Ward Hill, MA, USA) for the citrate complexation method. A homogeneous solution was obtained by mixing the zirconium precursor with the citric acid (1:1.2 molar ratio) in water. Zirconium ethoxide (Sigma Aldrich, St. Louis, MO, USA) was chosen as a precursor for this rapid hydrothermal method. The zirconium precursor was rapidly added as a powder in boiling water in order to obtain zirconium oxide nanoparticles. All the intermediate products were dried in air at 70 °C overnight. Impregnation of 1g ZrO_2_ with a 0.004 M solution of EuCl_3_·6H_2_O (Fluka, Waltham, MA, USA) with 10% Eu (OW and RH) or 20% Eu (CIT). The suspensions were stirred for 12 h at 60 °C, and then the separated solid was dried for 4 h at 80 °C under vacuum. Samples were calcined at 500 and 1000 °C in air with a heating/cooling rate of 10 °C/min and kept for 4 h at the maximum anneal temperature

The Brunauer–Emmett–Teller (BET) method was used to calculate the surface area from the data obtained at P/P_0_ (P = partial vapor pressure of adsorbate gas in equilibrium with the surface at 77.4 K; P_0_ = saturated pressure of adsorbate gas) between 0.01 and 0.995 sizes. Surface area (BET) measurements were conducted on precalcined samples (at 450 °C).

### 2.2. Compositional, Structural, and Morphological Characterization

The SEM micrographs and EDX spectra were acquired using an FEI Inspect S Electron Scanning Microscope (FEI, Hillsboro, OR, USA). Microbeam X-ray fluorescence (micro-XRF) spectrometry was performed on a custom-made instrument with an X-ray tube: Oxford Instruments (Abingdon, United Kingdom), Apogee 5011, Mo target, focus spot ≈40 µm, max. high voltage 50 kV, max current 1 mA, Amptek X-123 complete X-Ray spectrometer with Si-PIN detector. The key element of the micro-XRF instrument is an X-ray polycapillary minilens (IfG-Institute for Scientific Instruments, Berlin, Germany) which provides a focal spot size on the sample of 15–20 µm. Powder X-ray diffraction (XRD) patterns were recorded on a Shimadzu XRD-7000 diffractometer (Shimadzu Corp., Kyoto, Japan) using Cu Kα radiation (λ = 1.5418 Å, 40 kV, 40 mA) at a scanning speed of 0.10 degrees/min in the 10°–90° 2Θ range. The crystallite size was estimated using the Scherrer equation. Raman spectra were acquired in the extended spectral region from 150 to 4000 cm^−1^. Raman analysis was carried out with a Horiba JobinYvon-Labram HR UV-Visible-NIR Raman Microscope Spectrometer (Horiba Ltd., Kyoto, Japan) using the excitation wavelengths at 488, 514, and 633 nm. For TEM measurements, samples were prepared by grinding them in a mortar, followed by ultrasonic dispersion in ethanol and drop casting on a TEM grid provided with a holey carbon membrane. The specimens were analyzed using two electron microscopes. The low-magnification and HRTEM images, as well as the EDS (energy dispersive X-ray spectroscopy) spectra, were recorded using a JEM 2100 analytical TEM (JEOL Ltd., Tokyo, Japan) operated at 200 kV.

### 2.3. Luminescence

The photoluminescence measurements were carried out at room temperature and T = 80 K (by use of exchange gas cryostat) using a Fluoromax 4 spectrofluorometer (Horiba Ltd., Kyoto, Japan) operated in both the fluorescence and the phosphorescence mode. For excitation spectra the integration window varied between 0.1 and 0.5 s, the slits were varied from 0.1 to 1 nm in excitation, and from 1 to 3 nm in emission. The emission decays were measured by using the “decay by delay” feature of the phosphorescence mode. The repetition rate of the xenon flash lamp was 25 Hz, the integration window was set to 10 ms, the delay after flash varied between 0.03 and 25 ms, and up to 30 flashes were accumulated per data point. The average decay lifetime was calculated as integrated area of normalized emission decay. Time-resolved (gated) emission spectra (TRES) were recorded at low temperature, T = 80 K (by use of exchange gas cryostat), using a wavelength tunable NT340 Series EKSPLA OPO (Optical Parametric Oscillator, EKSPLA, Vilnius, Lithuania) for samples excitation (210–2300 nm), operated at 10 Hz. The tunable wavelength laser has a narrow linewidth (<5 cm^−1^, which makes the laser a high selective excitation source) with a scanning step and output energy depending on the spectral region. As a detection system, an intensified CCD (iCCD) camera (Andor Technology Ltd., Belfast, Ireland) coupled to a spectrograph (Shamrock 303i, Andor, Belfast, Ireland) was used. The TRES were collected using the box car technique. The gain of the micro-channel plate (MCP gain) was set to 100. The emission was detected in the spectral range of 500 nm < λ_em_ < 750 nm with a spectral resolution from 0.05 to 0.45 nm and the input slit of the spectrograph was set to 10 µm with a delay after the laser pulse varying from a few µs to 40 ms. The temperature of the iCCD was lowered to −20 °C for a better signal to noise ratio (S/N). For all measurements done using the iCCD, cut off filters were used to protect the detector from the excitation light.

## 3. Results

### 3.1. Assessment of Solid Solution Homogeneity by X-Ray Diffraction, Raman Spectroscopy, and Transmission Electron Microscopy

Throughout the text, the impregnated ZrO_2_ with 10% or 20% Eu are labelled as 10Eu(I)–ZrO_2_ (OW), 10Eu(I)–ZrO_2_ (RH), and 20Eu(I)–ZrO_2_ (CIT), where OW, RH, and CIT refer to oil in water microemulsion, rapid hydrothermal, and citrate complexation methods, respectively, used in the synthesis of bare ZrO_2_ nanoparticles. Oil in water microemulsion proved to be superior to the water in oil microemulsion method since the major (continuous) phase was water, while keeping the great advantages of the microemulsion method for the particle preparation (excellent control of nanoparticle characteristics such as size, composition, good crystallinity, high-surface area, etc.). However, this method has a few disadvantages due to separation and numerous washing steps and use of different oil phases that lead to low reproducibility [41,42]. The rapid-hydrothermal synthesis method assumes the immersion of alkoxide precursor powders into boiling water [40]. The advantages of this experimental method relate to the simple experimental setup and easily controllable particle size in time. Low reproducibility of the products due to their high dependence on the experimental conditions and the fact that high reaction rates cannot be expected represent important disadvantages of this procedure. Sol-gel citrate produces homogeneous nanopowders that involve the growth of metal oxo-polymers in a solvent. The sol-gel process involves hydrolysis and condensation, which are generally fast and need to be inhibited to avoid precipitation and allow sol or gel formation [43]. Simple synthesis steps and good reproducibility of this method are its main characteristics; however, particle agglomeration may be significant. All methods deliver amorphous particles with small, below 10 nm crystallite sizes. Surface area (BET) measurements on precalcined samples (at 450 °C) delivered values of few m^2^/g (CIT), and up to 250–300 m^2^/g for OW and RH samples. Prior to investigations, the bulk elemental composition of 10% (OW and RH) and 20% impregnated ZrO_2_ (CIT) was measured using EDX-SEM and X-ray fluorescence.

**Figure 1:**
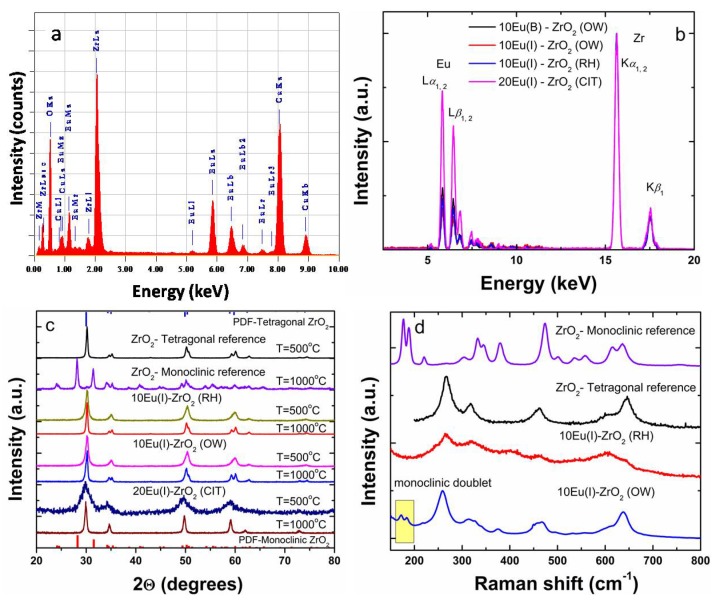
(**a**) Elemental chemical analysis using EDS (10Eu–ZrO_2_(OW)). The C and Cu peaks were present due to the copper grid provided with a carbon membrane; (**b**) XRF spectra of 10Eu(B)–ZrO_2_ (OW), 10Eu(I)–ZrO_2_ (OW), 10Eu(I)–ZrO_2_ (RH) and 20Eu(I)–ZrO_2_ (CIT); (**c**) XRD patterns, annealing temperatures of 500 and 1000 °C; and (**d**) selected Raman spectra, annealing temperature of 1000 °C.

As shown in Figure 1a,b and Table 1, the estimated values were in good agreement with the nominal concentration determined from precursors. Figure 1c gathers the XRD patterns of 10Eu(I)–ZrO_2_ (OW), 10Eu(I)–ZrO_2_ (RH), and 20Eu(I)–ZrO_2_ (CIT) following annealing in air at 500 and 1000 °C. Except for the 20Eu(I)–ZrO_2_ annealed at 500 °C, all patterns display a pure tetragonal phase (04-013-4748) (space group P4_2_/nmc). Within the instrumental sensitivity limit of XRD of a few %, the presence of additional impurity phases (e.g., ZrO_2_ monoclinic-PDF card: 00-036-0420) was not detected. Apparently, homogenous solid solutions were being formed even following mild calcination at 500 °C (Figure 1c). In the case of 20Eu(I)–ZrO_2_ (CIT), the broadened X-ray diffraction patterns due to the small crystallite size (around 3 nm) hindered the discrimination between cubic and tetragonal phases, as the tetragonal (P4_2_/nmc) phase is a slightly distorted variant of the cubic phase ($F_{m\bar{3}m}$) [1]. As listed in Table 1, the crystallite sizes estimated using the Scherrer equation increased from 13 nm and 15 nm to 23 nm and 17 nm for the 10Eu(I)–ZrO_2_ (OW) and 10Eu(I)–ZrO_2_ (RH), respectively, and from a few nm to 16 nm for 20Eu(I)–ZrO_2_ (CIT) with the increase of the annealing temperature from 500 to 1000 °C.

Selected Raman spectra following annealing at 1000 °C are gathered in Figure 1d. To eliminate any interference from the relatively more intense luminescence lines of Eu, the Raman spectra were measured at three excitation wavelengths (488, 514 and 633 nm). For comparison purposes, the Raman spectra of the monoclinic and tetragonal ZrO_2_ (RH support annealed at 1000 and 500 °C, respectively) used as references, are also included. The crystal structure of tetragonal ZrO_2_ is a body-centered lattice with the space group P4_2_/nmc with Zr(4+) eight fold coordinated to oxygen atoms in D_2d_ point group symmetry. Raman spectra show five of the six fundamental optical modes in the range of 200 to 700 cm^−1^, namely at 265 cm^−1^ (B_1g_), 318 cm^−1^ (E_g_), 470 cm^−1^ (E_g_), and 649 cm^−1^ (A_1g_), at similar positions regardless of the excitation wavelength and therefore assigned unambiguously to the Raman bands of tetragonal ZrO_2_ [44]. For the OW sample, a small contribution from the monoclinic phase via the phonon band doublet modes Bg and Ag at 178 and 190 cm^−1^, respectively, that were not overlapped with any tetragonal bands was clearly observed. Additional phonon modes characteristic of Eu_2_O_3_ C-phase were not detected in any of the impregnated samples.

**Figure 2:**
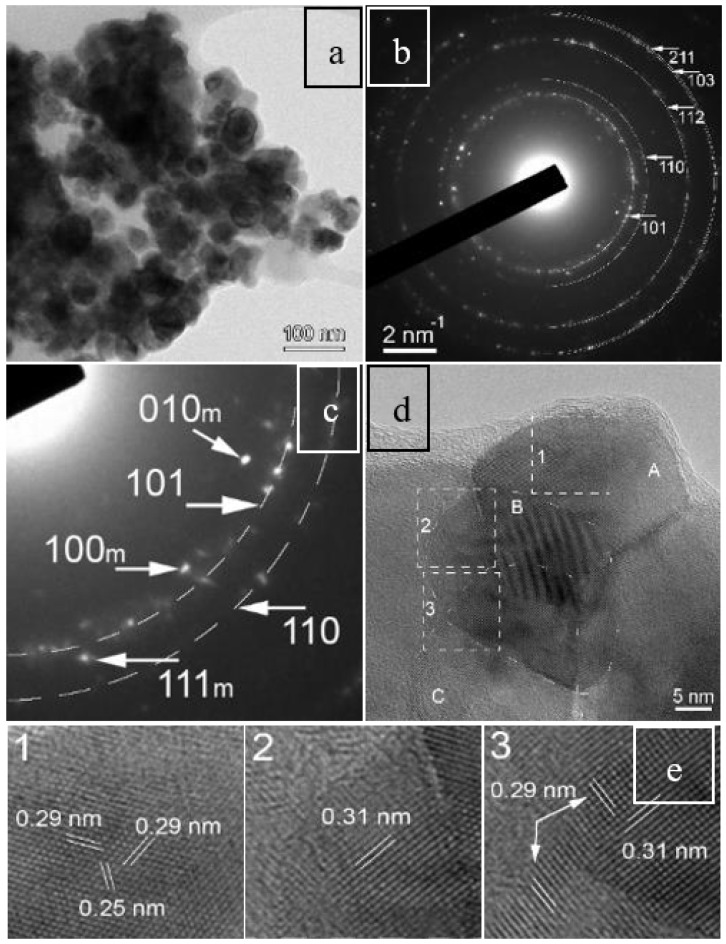
(**a**) TEM image showing typical wet impregnated 10Eu(I)–ZrO_2_ (OW) nanoparticles with a size below 50 nm. (**b**,**c**) Corresponding SAED pattern and an enlarged part of it proving the formation of the ZrO_2_ tetragonal dominant phase and of a minor monoclinic phase. (**d**) HRTEM image and (**e**) zoomed-in images from three different areas confirming the occurrence of a minor monoclinic phase.

Due to the small content of monoclinic content detected using Raman spectroscopy, we selected the OW sample (1000 °C annealing) for TEM characterization. As shown in Figure 2a, a relatively narrow range of particle sizes was measured, ranging from 20 to 40 nm, which suggested a mild agglomeration when compared with the values of crystallite sizes estimated from XRD (Table 1). The selected area electron diffraction (SAED) pattern exhibited a mixture of crystallographic structures with tetragonal and monoclinic ZrO_2_ as a major and minor phase, respectively (Figure 2b,c). In Figure 2b, half rings have been drawn through the scattered diffraction spots of the tetragonal phase, indicating also the corresponding Miller indices, in agreement with the PDF Card 88–1007 of tetragonal ZrO_2_. A series of spots were observable next to these rings that were related to the monoclinic phase of ZrO_2_ in agreement with the PDF card 88–2390 (Figure 2c). The formation of the secondary monoclinic phase was also confirmed using high-resolution TEM images in Figure 2d. Lattice parameters and overall scale-factor were determined by refinement of whole powder pattern fitting, which allowed the tuning of lattice parameters via iterations in order to find a fit with known lattice parameters [45].

The quantitative analysis of the EDS spectra revealed some non-uniformity in Eu local concentrations that vary from as low as 2.2 at% to 9.2 at%, which explains the occurrence of the monoclinic and tetragonal polymorphs. The minor monoclinic phase of impregnated 10Eu(I)–ZrO_2_, disregarded by XRD (Figure 1b) but detected by Raman spectroscopy (Figure 1c), could also be revealed by HRTEM in a nice example of techniques complementarity.

### 3.2. Use of Eu Luminescence as a Local Probe

#### 3.2.1. Overview of the Luminescence Properties

**Figure 3:**
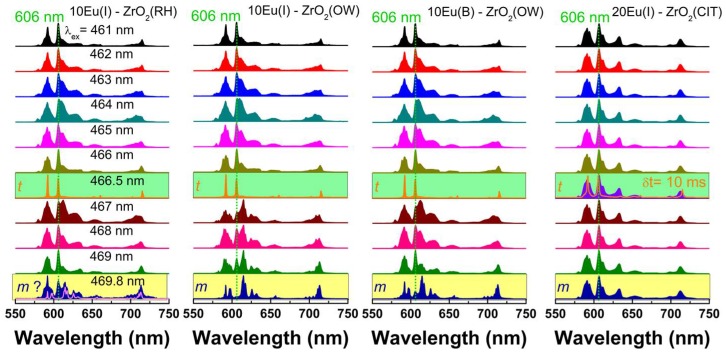
Comparison between the site-selective emission spectra of 10Eu(I)–ZrO_2_ (OW, RH) and 20Eu(I)–ZrO_2_ (CIT) performed at low temperature (T = 80 K). For comparison only, a selection of emission spectra of 10% Eu bulk doped using the OW method and labelled as 10Eu(B)–ZrO_2_(OW) is also included. The dotted green line highlights the appearance of the tetragonal fingerprint emission (defined by the 606 nm peak). The green highlighted spectra correspond to well-separated tetragonal emission. The yellow highlighted spectra represent the contribution from the monoclinic fingerprint mission.

**Figure 4:**
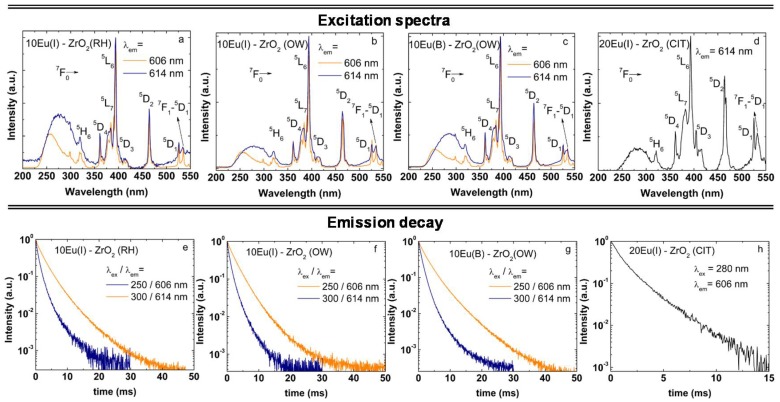
Comparison between excitation spectra and emission decays of 10Eu(I)–ZrO_2_ (OW, RH) and 20Eu(I)–ZrO_2_ (CIT). For comparison only, the excitation spectra and emission decays of 10Eu(B)–ZrO_2_(OW) are also included. To separate the tetragonal and monoclinic contributions, the monitored emission wavelengths were set at 606 and 614 nm, while the excitation wavelengths were set at 250 and 300 nm. All samples were annealed at 1000 °C. The measurements were performed at room temperature.

The main structural difference between monoclinic, tetragonal, and cubic phases of zirconia is given by the displacements of the lattice oxygen atoms; therefore, consideration of the local atomic structure is essential to fully understand the effect of doping on ZrO_2_ [3,4]. An effective approach for local structure investigations is based on Eu luminescence, which is highly sensitive to local symmetry. The local symmetry at the Eu site varies from low C_2_, (Cs or C_1_) symmetry in seven-fold coordination in monoclinic phase, to the higher symmetry (eight-fold coordination) of tetragonal (D_2d_) or cubic (O_h_) phases. The emission properties of Eu in fully stabilized tetragonal/cubic ZrO_2_ (all obtained exclusively by bulk doping methods) are well documented in the literature and the reader can refer to References [6,10,11,15,16,17,18,19,20,25,27,33,35,36,38,46,47,48]. Usually, in these studies, the excitation was performed using UV that contained the absorption profiles of tetragonal ZrO_2_ [1] and O^2−^–Eu oxygen charge transfer [49] or f-f absorption transitions of Eu at 394 and 464 nm. To check on the homogeneity at the atomic scale of 10Eu(I)–ZrO_2_ (OW, RH) and 20Eu(I)–ZrO_2_ (CIT) (all samples at 1000 °C annealing), we have performed extensive low-temperature (80K), site selective, time-gated excitation spanning the UV to visible spectral range. A selection of emission/excitation spectra obtained under excitation across the ^7^F_0_-^5^D_2_ hypersensitive transition around 464 nm is illustrated in Figure 3 and Figure 4.

Several general characteristics emerge from the comparison of emission spectra in Figure 3: (1) The emission shape characteristic of the ^5^D_0_ level of Eu changes with the excitation wavelength while the associated width varies from 1 to 10 nm, which sustains the coexistence of both ordered and disordered local environments. (2) The spectral feature around 606 nm characteristic of Eu substituting for Zr in tetragonal sites [17,18,19,27,30,49] (with no defect nearby or not locally charge-compensated) can be observed at selected wavelengths. In tetragonal ZrO_2_, the eight-folded Zr(4+) has two sets of short (2.17 Å) and long (2.36 Å) Zr–O bond lengths, which define a D_2d_ inversion, that is a less symmetrical environment around Zr [3]. The well-known emission fingerprint of Eu in tetragonal zirconia is given by two emissions bands of comparable intensity at 591 nm (corresponding to the merging of the two permitted ^5^D_0_-^7^F_1_ transition lines) and ≈606 nm (which is the strongest among the four allowed ^5^D_0_-^7^F_2_ lines), (the green highlighted spectra in Figure 3). (3) Basically, there are no differences observed between the emission shapes when comparing the wet impregnated and bulked doped samples (OW). (4) The excitation modes and emission dynamics are similar across OW and RH samples (Figure 4). This means that not only the electronic interactions between the dopant and zirconia host (excitation spectra), but also the balance between the radiative (local symmetry) and non-radiative transition probabilities (due to interaction between Eu ions or between Eu with structural and surface defects) are similar across the three samples. (5) For the 20Eu(I)–ZrO_2_(CIT), the Eu local environment is remarkably homogenous, as shown by the constancy of the emission shape with the excitation wavelength. The emission is broader as compared to typical emission of RH and OW samples, as a result of the enhanced amount of oxygen vacancies generated by the higher Eu concentration. Most of these apparently locate near to Eu, since the fingerprint emission of Eu in tetragonal sites at 606 nm can be separated only under time-gated emission conditions (using at least 10 ms delay after the laser pulse).

#### 3.2.2. Assessment of Solid Solution Homogeneity at the Atomic Scale. Comparison with CeO_2_

We further checked on the homogeneity at the atomic scale of the impregnated tetragonal zirconia samples. More specifically, we looked for the sources that might interfere with Eu emission substituting for Zr lattice sites identified as (1) Eu in minor monoclinic phase, (2) segregated Eu as Eu_2_O_3_ phase, (3) Eu–Zr pyrochlore oxide as Eu_2_Zr_2_O_7_ [50], and (4) segregated Eu as surface Eu.

(1) A small amount of the monoclinic content is determined using Raman spectroscopy only for the OW sample (Figure 1c). In luminescence, the contribution of the monoclinic-type emission to overall luminescence spectra is clearly detected in both RH and OW samples via its fingerprint emission [18,49]. Eu emission in monoclinic sites is dominated by the ^5^D_0_–^7^F_2_ electric dipole transition centered at 614 nm. In the spectral range of ^5^D_0_–^7^F_1_ transition, one line at ≈591 nm and a doublet of closely spaced lines at 597 and 598 nm represent the signature for the monoclinic emission. Since the characteristic luminescence of monoclinic phase is measured also in the bulk doped counterpart, 10Eu(B)–ZrO_2_ (OW), it appears that such “incomplete” tetragonal phase stabilization is not favored using the wet impregnation route. It is also observed that the contribution of monoclinic emission to overall emission is rather strong, despite all samples being purely tetragonal, as seen in the XRD. This is likely due to larger radiative emission rates of europium in the low, monoclinic symmetry (C_1_) compared to more symmetrical (D_2d_) tetragonal sites [51]. (2) Usually, the identification of segregated Eu_2_O_3_ is strongly dependent on the instrument type and sensitivity, as well as the synthetic approach, which determines the dopant distribution homogeneity. It is highly probable that upon impregnation, some of Eu segregate as cubic C-type Eu_2_O_3_ ($I_{a\bar{3}}$). The amount of this parasite phase may be small enough to remain undetected by XRD and Raman spectroscopy (due to the instrumental sensitivities) as well as TEM (due to the limited volume of the analyzed sample). However, the measured emission of cubic Eu_2_O_3_ (obtained by calcining the as received Eu(NO_3_)_3_·xH_2_O (x≈6) from Alfa Aesar at 1000 °C) using several excitation wavelengths resembles the characteristic emission of Eu in Y_2_O_3_ [52] (spectra not included) distinct to any of the spectral shapes of impregnated zirconia illustrated in Figure 3. (3) The same conclusion is drawn considering the emission shapes of Eu characteristic of Eu_2_Zr_2_O_7_ of disordered fluorite or ordered pyrochlore structures reported in the literature [53]. (4) Finally, the presence of a significant surface Eu, that is typically characterized by a short-lived, strongly distorted emission [54] is ruled out in all impregnated samples by use of time-gated luminescence.

As shown in Figure 4e–h, the emission decays are in the ms range, suggesting that Eu substitute for Zr in the inner lattice sites. The estimated average lifetimes of ^5^D_0_ level monitoring emissions at 606 and 614 nm (Table 2) are very similar for the RH and OW samples (2.45–2.76 ms and 0.93–1.07 ms, respectively). For 20Eu(I)–ZrO_2_(CIT), the lifetime corresponding to the emission peaked at 606 nm is slightly longer at 1.3 ms.

It should be noted that the ability of a nanosized fluorite oxide of dissolving high amount of lanthanide oxides (up to 30%) by wet impregnation was first suggested by Corma et al. [55] for the fluorite-structured oxide of Ce (CeO_2_) and confirmed in subsequent studies by some of us [56,57]. We note that the tetragonal structure (along with the monoclinic one) originates from the parent fluorite (CaF_2_) structure [4]. However, fluorite ZrO_2_ differs from fluorite CeO_2_, both in the size differences of the lattice structure [58], and ionic radii mismatch as the lanthanide are significantly bulkier relative to Zr than the Ce host cation (ionic radii of Eu = 1.066 Å, Zr = 0.84 Å, and Ce = 0.97 Å in eight-fold coordination) [59]. Therefore, we initially expected that ZrO_2_ is less efficient in dissolving Eu oxide using wet impregnation and more prone to phase separation. Still, preliminary XRD and luminescence data indicates that wet impregnation of ZrO_2_ with 20% Eu is leading to a homogenous solid solution following annealing at 500 °C (as confirmed by XRD) and 1000 °C (as confirmed by XRD and luminescence). Apparently, such behavior is due to the common ability of fluorite-structured oxides to accommodate a large amount of oxygen vacancies [60]. The size mismatch between the lanthanide dopant and Zr host cation does not represent a limiting factor in obtaining homogenous doped zirconia solid solutions via wet impregnation.

## 4. Conclusions

Wet impregnation is a well-known method used for synthesis of heterogeneous catalysts, that is, surface doped oxides. Here, we show that wet impregnation with 10 and 20% Eu followed by calcination in air above 500 **°C** produces a full tetragonal phase stabilization of ZrO_2_ nanoparticles in the bulk. The preformed ZrO_2_ nanoparticles were obtained using three synthetic routes: oil in water microemulsion, rapid hydrothermal, and citrate complexation. First, the homogeneity of Eu-doped tetragonal solutions was assessed using X-ray diffraction, Raman spectroscopy, and transmission electron microscopy. The homogeneity at the atomic scale was further confirmed using Eu luminescence as a local probe under time-resolved, site-selective excitation conditions at room-temperature and 80 K. We believe that wet impregnation will allow the first systematic and reproducible set of investigations on the structural properties of ZrO_2_ doped with fixed trivalent lanthanide at various concentrations as well as with trivalent lanthanides across the whole 4f series at a fixed concentration.

## Figures and Tables

**Table 1:** Crystallite size and cell parameters of 10Eu(I)–ZrO_2_ (OW, RH) and 20Eu(I)–ZrO_2_ (CIT) following annealing at 500 and 1000 °C. Bare ZrO_2_ nanoparticles were amorphous prior to annealing, irrespective of the synthetic route.

	Crystallite Size (nm) Calculated from XRD, (±0.5 nm) and Cell Parameters (±5 × 10^−4^ Å)						Eu Concentration Estimated from XRF Data (±1.5%)
**Sample**	500 °C	c	a	1000 °C	c	a	
**10Eu(I)-ZrO_2_(RH)**	13	5.135	3.626	23.3	5.185	3.606	9.6%
**10Eu(I)-ZrO_2_(OW)**	15	5.130	3.627	16.5	5.194	3.608	9.1%
**10Eu(B)-ZrO_2_(OW)**	14.4	5.189	3.613	16.2	5.200	3.614	10.2%
**20Eu(I)-ZrO_2_(CIT)**	*	-	-	16.5	5.220	3.673	21.4%

*—XRD reflections were too broad to estimate whether Eu stabilizes the tetragonal/cubic phase or eliminate the presence of parasite phases.

**Table 2:** Average lifetimes of 10Eu(I)–ZrO_2_ (OW, RH) and 20Eu(I)–ZrO_2_ (CIT) annealed at 1000 °C estimated from the emission decays displayed in Figure 4e–h.

Sample	Estimated Average Lifetime (ms) (±0.01 ms)	
	λ_ex_ = 250 nm λ_em_ = 606 nm	λ_ex_ = 300 nm λ_em_ = 614 nm
**10Eu(I)–ZrO_2_(RH)**	0.97 ms	2.47 ms
**10Eu(I)–ZrO_2_(OW)**	0.93 ms	2.45 ms
**10Eu(B)–ZrO_2_(OW)**	1.07 ms	2.76 ms
**20Eu(I)–ZrO_2_(CIT)**	* 1.3 ms	

* For 20Eu(I)–ZrO_2_(CIT), the average lifetime was estimated from emission decay excited at 280 nm and monitored at 606 nm.
